# Tetris Genioplasty 2.0: The Evolution of the Technique

**DOI:** 10.3390/jcm13216503

**Published:** 2024-10-30

**Authors:** Valerio Ramieri, Linda Latini, Guido Gabriele, Vittoria Fantozzi, Tito Matteo Marianetti, Flavia Cascino

**Affiliations:** 1 Ortognatica Roma, Via Nomentana 311, 00137 Roma, Italy; 2 Maxillofacial Surgery Unit, Department of Mental Health and Sense Organs, Azienda Ospedaliera Universitaria Senese, 53100 Siena, Italy

**Keywords:** genioplasty, orthognathic surgery, chin genioplasty, asymmetry surgery

## Abstract

The chin is a key feature in facial aesthetics, contributing essential projection to the profile. Proper alignment ensures facial harmony, while misalignments can create irregularities that may require surgical correction. Over time, various genioplasty techniques have been developed to address a broad range of asymmetries. Tetris Genioplasty is a novel osteotomy technique designed to improve cases where the skeletal midline does not align with the dental midline, delivering significant corrective results. Building on extensive experience with patients presenting this condition, this technical note introduces Tetris Genioplasty 2.0, a refined version of the original “Tetris” Osteotomy. This enhanced technique minimizes the presence of edges during bone segment realignment, resulting in a more seamless facial profile. This study aims to detail the advancements in the Tetris Genioplasty technique, demonstrating how the 2.0 modification offers an optimized approach to improving facial symmetry and aesthetic outcomes in complex midline discrepancies.

## 1. Introduction

The correction of chin irregularities poses a significant challenge for maxillofacial surgeons, who must adopt a personalized approach for each patient. This is particularly true in cases where the chin structure is asymmetric, a condition that can disrupt the harmony of the face, causing both aesthetic and functional discomfort [1]. In recent years, the rise of orthodontic compensation treatments has led to an increase in patients who, despite having a centered dental midline, still exhibit facial asymmetry [2]. This discrepancy between the dental alignment and the skeletal structure of the face has highlighted the need to develop new techniques to more effectively and harmoniously address chin irregularities.

One innovative technique that meets this need is Tetris Genioplasty [3], an osteotomy designed specifically to treat chin asymmetry. Traditionally, chin asymmetry has been corrected by moving the chin tip in three dimensions, but this approach has its limitations [4]. If the movement is limited to the chin tip, discontinuities can appear along the mandibular border, leading to less harmonious aesthetic outcomes. The Tetris Genioplasty technique aims to overcome these challenges, allowing for a more comprehensive and precise correction of chin asymmetries. The procedure begins with an incision made approximately 15 mm from the mucogingival line, followed by a subperiosteal dissection of the mentalis muscle. A rectangular osteotomy is then performed, similar to that used in telescopic genioplasty [5]. The transverse limit of this osteotomy is determined by the intercanthal distance, while the height typically ranges from 15 to 20 mm from the lower border of the mandible, considering the proximity of the tooth roots. However, unlike telescopic genioplasty, Tetris Genioplasty includes an additional vertical osteotomy, where the width corresponds to the distance between the skeletal midline and the dental midline.

Once these osteotomies are completed, two separate bone blocks are obtained. The smaller block is removed, creating an empty space that will be filled by the larger block, which is translated into the new position. After this translation, the remaining empty space on the opposite side is filled by the smaller block. This exchange of positions between the bone blocks resembles the fitting of pieces in a game of Tetris, hence the technique’s name. This method allows for a more comprehensive correction of the asymmetry, addressing not only the position of the chin tip but also its relationship with the overall mandibular structure.

Despite the advantages of Tetris Genioplasty, in some patients, especially those presenting with both chin and mandibular asymmetry, irregularities can appear along the mandibular border. These patients, who exhibit asymmetry in the chin and the mandible, may experience less harmonious final results. In such cases, correction limited to the chin alone might lead to the appearance of sharp edges along the mandibular border, compromising the overall aesthetic of the face. To address this potential complication, the authors of the technique have developed an enhanced version called Tetris 2.0, which aims to avoid the formation of sharp edges and ensure a smoother, more natural mandibular contour.

Tetris 2.0 introduces some key modifications compared to the original technique. In addition to translating the bone blocks to their new positions, Tetris 2.0 involves more detailed reshaping of the mandibular border, paying special attention to the transitions between various parts of the mandible and chin. This evolution of the technique allows for more harmonious results, especially in patients with complex asymmetries.

## 2. Materials and Methods

In 2023, one of the authors successfully performed five Tetris Genioplasty 2.0 procedures. The inclusion criteria for these cases were based on the presence of mandibular asymmetries on the frontal plane, specifically in patients with a misalignment between the dental midline and the skeletal midline that would not have been effectively addressed by the classic Tetris Genioplasty technique. These patients presented with aesthetic concerns only, with no reported functional impairments related to phonation, swallowing, or breathing. Table 1 summarizes the patients’ data. As part of the surgical protocol, all patients signed informed consent documents in accordance with the ethical principles outlined in the Declaration of Helsinki, also providing permission for the use of their data and images in scientific publications. Ethical approval for these procedures was obtained from the Institutional Review Board (IRB) of the University of Siena, with the assigned approval number 9/2021. Before surgery, all patients underwent a Cone-Beam Computed Tomography (CBCT) scan, allowing for detailed craniofacial structure imaging. The CBCT data were saved in the standard Digital Imaging and Communications in Medicine (DICOM) format and then imported into Dolphin Imaging Software 12.0, an advanced software suite specifically designed for virtual surgical planning (VSP) in orthognathic procedures. Using this software, the DICOM images were reformatted into 3D STL files, enabling a highly detailed and precise visualization of the anatomy, essential for planning the intricate details of the procedure. Each patient was photographed from multiple angles (frontal, oblique, basal, and lateral views) both before the surgery and during each postoperative visit to monitor progress and outcomes.

The surgical procedure itself was performed under general anesthesia, with orotracheal intubation ensuring secure airway management. The initial phase of the Tetris Genioplasty 2.0 mirrors the steps of the original technique. An incision was made using an electric scalpel approximately 15 mm from the mucogingival margin, followed by careful subperiosteal dissection of the mentalis muscle to expose the underlying bone of the chin. A rectangular-shaped osteotomy was then executed, with the transverse limits aligned with the patient’s intercanthal distance and the vertical height ranging between 15 and 20 mm from the lower mandibular border. A second vertical osteotomy was also performed, with the width precisely matching the discrepancy between the skeletal midline and the dental midline.

This is where Tetris Genioplasty 2.0 distinguishes itself from the classic approach. After the two bone blocks were mobilized, the smaller block was removed. The larger block was then translated into the space left by the smaller one, correcting the chin asymmetry. However, in contrast to the original Tetris Genioplasty technique, the smaller bone block was not repositioned on the opposite side after its removal.

**Figure 1:**
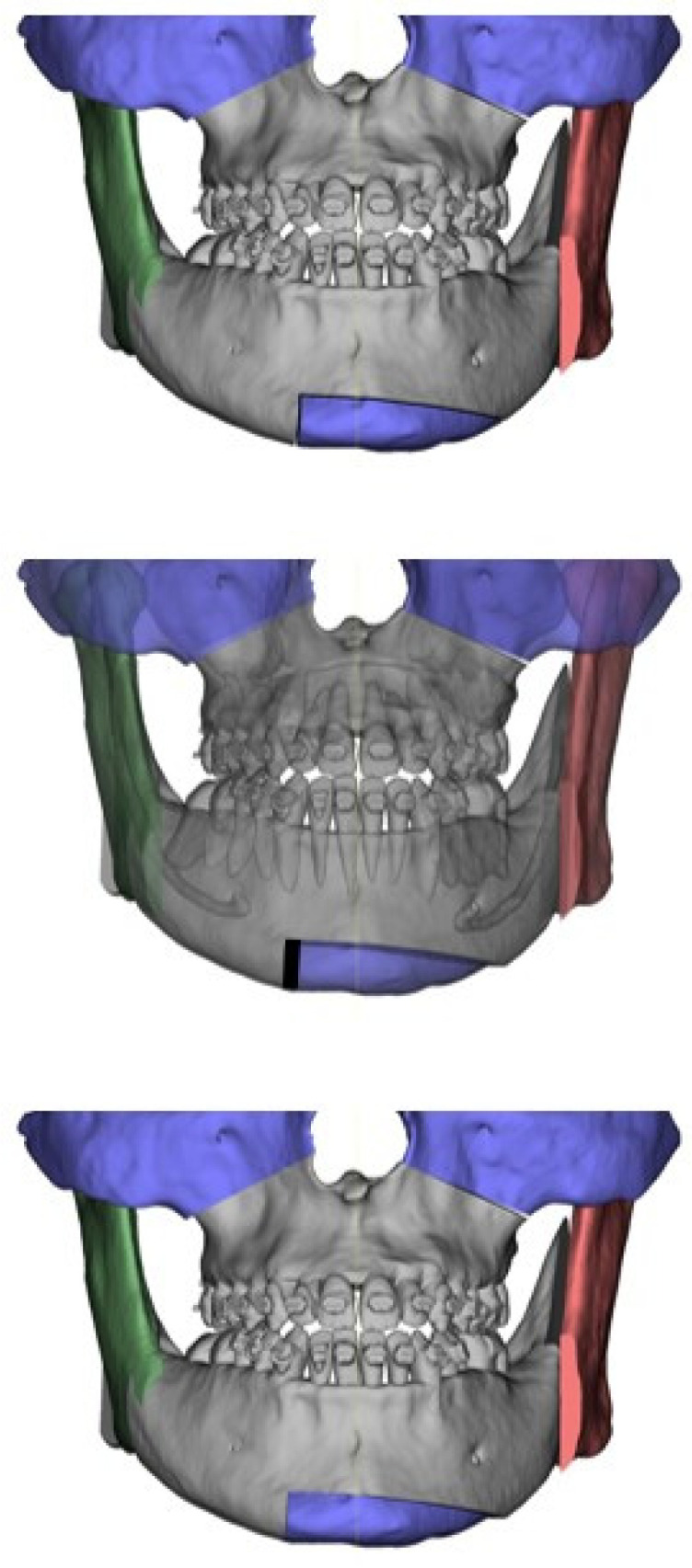
Surgical planning.

Figure 1 represents a schematic diagram of the technique.

This modification not only simplified the procedure but also reduced the risk of irregularities and unnecessary disruptions along the mandibular contour. Once the osteotomies were complete, the bone fragments were securely fixed in place using plates and screws to ensure stability during the healing process.

Following the surgery, each patient remained in the hospital for a 24 h observation period to monitor initial recovery. During the postoperative period, we administered antibiotics (Amoxicillin + Clavulanic Acid 1 g three times a day for 7 days) and analgesics as needed. No patient required analgesics for more than 5 days. Also, in the postoperative phase, the health and viability of the lower incisors were assessed using an electronic pulp tester, a device that checks the responsiveness of the tooth pulp to ensure no nerve damage occurred during the procedure. The average follow-up period was 6 months, with a range extending from 3 to 8 months depending on individual patient needs.

Follow-up care was highly personalized, beginning with a clinical examination one-week post-surgery, followed by additional assessments at the 3-month and 6-month marks. In cases where patients demonstrated excellent clinical recovery, the follow-up period could be shortened. Conversely, for patients requiring more attention due to slower healing or other factors, the follow-up period was extended to ensure comprehensive care. Throughout the entire process, the focus was on maintaining patient safety and achieving optimal aesthetic results, ensuring the success of this innovative surgical technique.

## 3. Results

**Figure 2:**
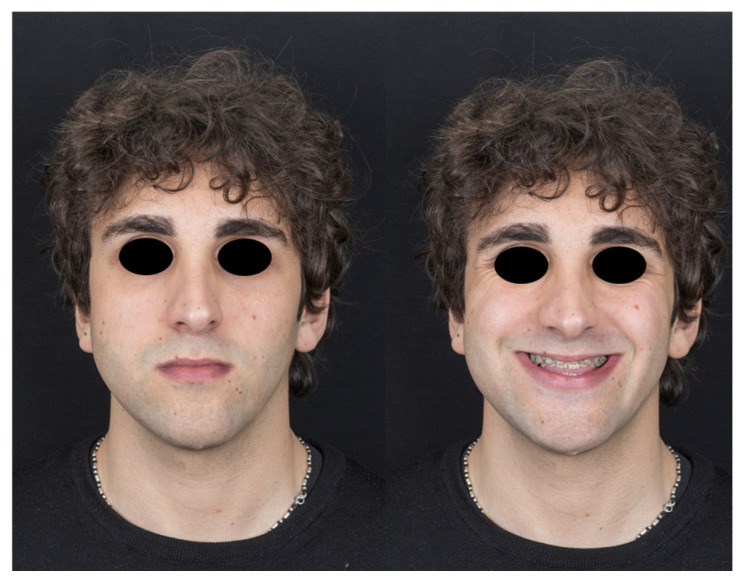
Preoperative appearance.

**Figure 3:**
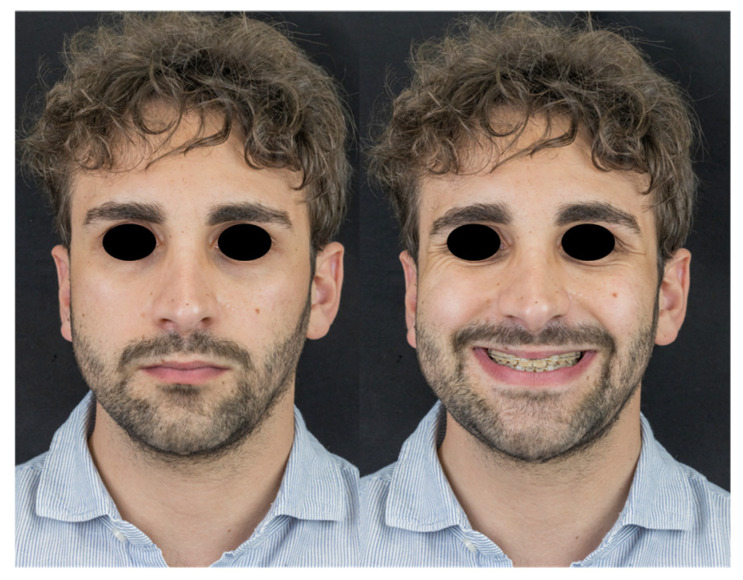
Postoperative appearance.

The preoperative and postoperative appearances of one patient are illustrated in Figure 2 and Figure 3, respectively.

Throughout the follow-up period, no postoperative complications were observed, such as infection, extrusion of plates or screws, non-union of bone segments, or residual chin asymmetry. Importantly, there were no detected changes in the vitality of the lower anterior teeth and there were no sensitive alterations, confirming the procedure’s safety regarding nerve and dental health.

The postoperative check-ups were all conducted by the surgeon who performed the procedures and by the orthodontist who had previously treated the patient.

All patients expressed a high degree of satisfaction with the surgical outcomes, noting that the results were in line with their preoperative expectations. We assessed patient satisfaction by interviewing them during their postoperative check-ups; we asked each patient to rate their satisfaction on a scale from one to ten, and the average score was 8.

Since this procedure was purely aesthetic and none of the patients reported any pre-existing functional impairments such as difficulty with speaking, swallowing, or breathing, we consider subjective facial analysis, or the patient’s perception of their appearance, as a critical and valuable parameter in evaluating the success of the surgery.

This focus on subjective evaluation aligns with the goals of aesthetic surgery, where the primary aim is to enhance the patient’s facial harmony and self-esteem. Given that the patients were seeking aesthetic improvements rather than functional corrections, their satisfaction and perception of facial symmetry take precedence in determining the overall success of the procedure. Consequently, we believe that patient-reported outcomes serve as a reliable and effective metric for assessing the impact of surgeries like Tetris Genioplasty 2.0, emphasizing the importance of subjective facial analysis in aesthetic interventions.

## 4. Discussion

Tetris Genioplasty 2.0 is an advanced variation of the widely recognized Tetris Genioplasty, specifically designed to achieve more harmonious and aesthetically pleasing results in patients where the misalignment between the skeletal midline and the dental midline is due not only to chin asymmetry but also to the asymmetry of the entire mandible. This distinction is crucial because, in cases where the mandibular asymmetry extends beyond the chin, performing the classic Tetris Genioplasty could potentially create uneven or disharmonious contours along the mandibular border, leading to less satisfactory aesthetic outcomes.

The Tetris Genioplasty 2.0 technique addresses this challenge by providing a more comprehensive solution that considers the entire mandible rather than focusing solely on the chin. By allowing the surgeon to manipulate and reposition bone blocks in a more precise and controlled manner, it ensures a smoother transition between the chin and the rest of the jawline. This results in a more balanced and natural-looking outcome, with a seamless mandibular contour that aligns both functionally and aesthetically with the patient’s facial structure.

As with all osteotomies, in “Tetris genioplasty”, it is important to consider that using both Piezosurgery and a saw will result in a small loss of bone tissue, leaving a slight vertical gap where segments join due to the thickness of the instruments. While this may seem like a limitation, the authors believe that simply being aware of this factor allows the surgeon to calculate and plan the osteotomy with this minor discrepancy in mind.

One of the key advantages of Tetris Genioplasty 2.0 is its efficiency. The procedure is relatively quick to perform, often requiring less operative time compared to more traditional orthognathic surgeries. Additionally, it is considered minimally invasive, as it requires smaller incisions and less extensive bone manipulation. This reduces both the surgical trauma to the surrounding tissues and the overall recovery time for the patient.

From a safety standpoint, the technique has proven to be highly reliable. By incorporating careful preoperative planning, often using virtual surgical planning (VSP) software, surgeons can precisely calculate the osteotomies and reposition the bone segments with minimal risk of complications [6]. The method is also considered to be reproducible, meaning that other surgeons can implement the same technique with consistent results. The standardized steps involved in the osteotomies, bone block repositioning, and fixation with plates and screws allow for predictable outcomes across different cases, making Tetris Genioplasty 2.0 a versatile and dependable option for correcting complex mandibular asymmetries.

In conclusion, Tetris Genioplasty and its updated version, Tetris 2.0, represent a significant advancement in the techniques for correcting chin asymmetries. These techniques offer surgeons a more versatile and comprehensive approach, enabling a more harmonious and natural correction of chin and mandibular irregularities, ultimately improving not only the aesthetic appearance but also the quality of life for patients.

This technique provides significant benefits in terms of aesthetic outcomes, patient recovery, and overall satisfaction.

## Figures and Tables

**Table 1:** Patients’ data.

	Age	Sex	Impairment Complained	Previous Orthodontic Treatment	Surgery Procedure	Deviation Between Skeletal Median and Dental Median	Health Status	Smoking Habit
Patient 1	27	Male	Aesthetic Impairment	Yes	Tetris Genioplasty 2.0	2.96 mm	Good	No
Patient 2	19	Female	Aesthetic Impairment	Yes	Tetris Genioplasty 2.0	1.80 mm	Good	Yes
Patient 3	34	Male	Aesthetic Impairment	Yes	Bimaxillary Procedure + Tetris Genioplasty 2.0	3.65 mm	Good	Yes
Patient 4	27	Male	Aesthetic Impairment	Yes	Bimaxillary Procedure + Tetris Genioplasty 2.0	3.15 mm	Good	No
Patient 5	24	Male	Aesthetic Impairment	yes	Tetris Genioplasty 2.0	2.92 mm	Good	No

## Data Availability

The original contributions presented in the study are included in the article, further inquiries can be directed to the corresponding author.
